# Supplementary material for: Loss of Ezh2 in the medial ganglionic eminence alters interneuron fate, cell morphology and gene expression profiles
Source: Front Cell Neurosci. 2024 Feb 14;18:1334244. doi: 10.3389/fncel.2024.1334244 (PMC10899338; doi:10.3389/fncel.2024.1334244)
Supplement: Supplementary file 1 [file Presentation_1.pdf]

Supplementary Figures & Legends

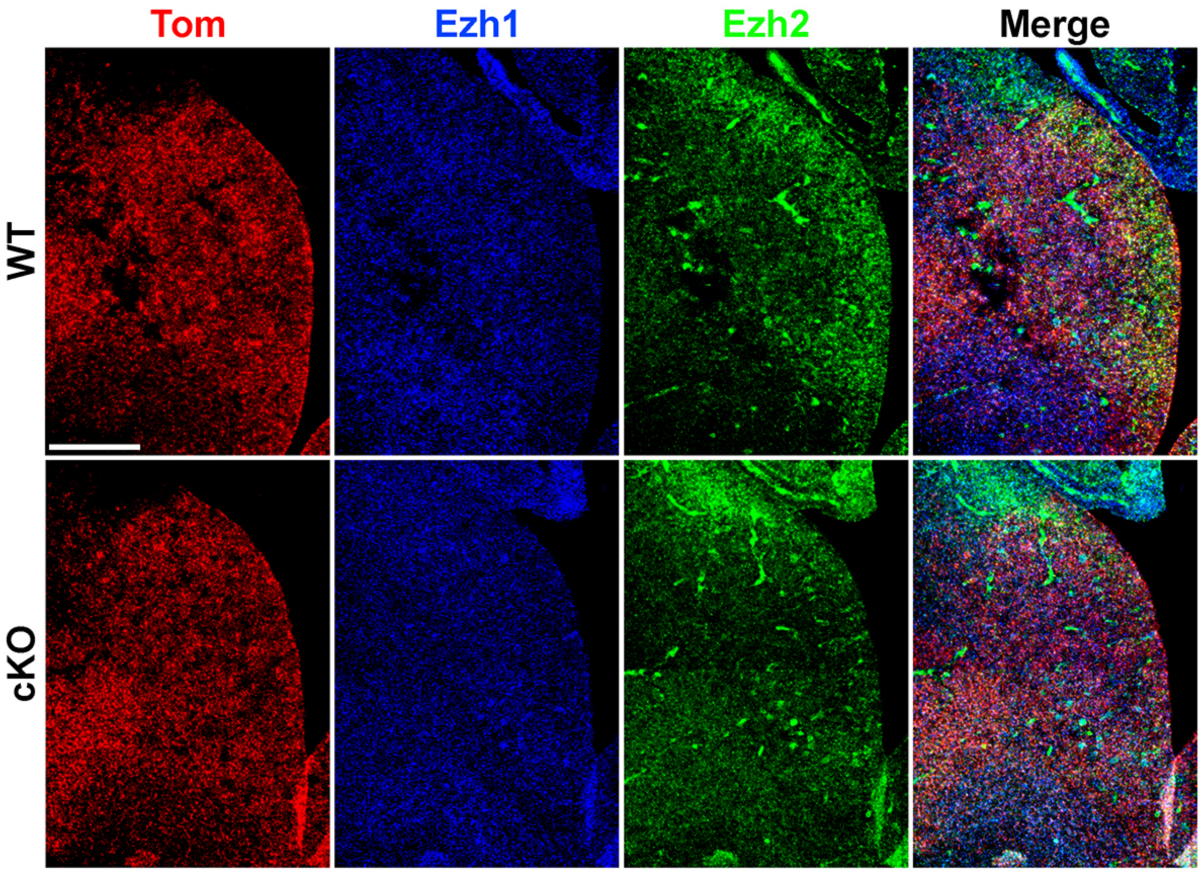

Supplementary Figure 1. Ezh1 expression in the MGE of WT and *Ezh2* cKO mice. *In situ* hybridizations depicting similar levels of Ezh1 mRNA in the MGE of *Ezh2* WT and cKO mice. Scale bar = 200 μm.

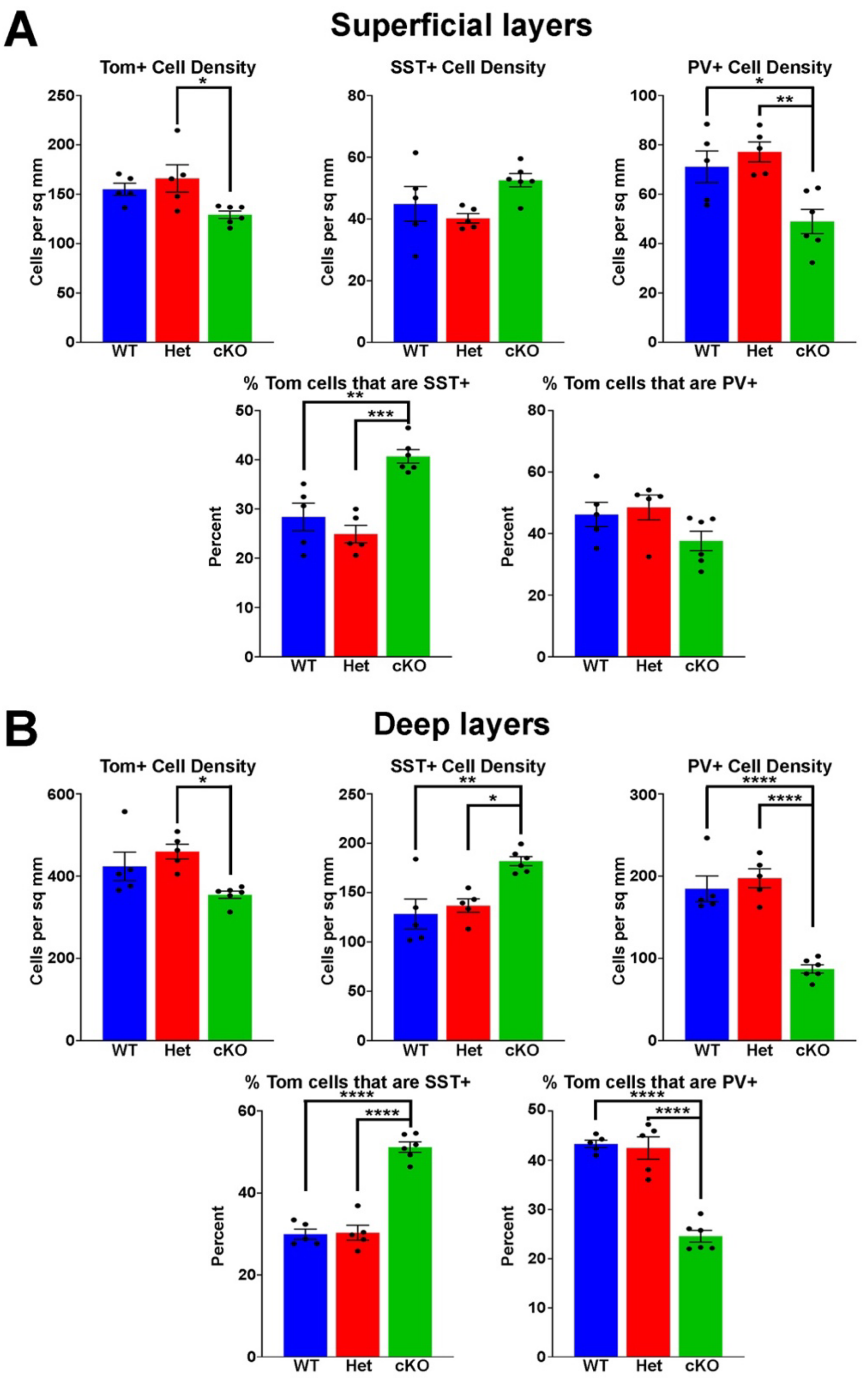

Supplementary Figure 2. Comparison of SST+ and PV+ interneurons in superficial and deep cortical layers. Graphs displaying the density of Tom+, SST+ and PV+ cells (top), and percent of Tom+ cells expressing SST or PV (bottom), for superficial (**A**) and deep (**B**) cortical layers. All stats are one-way ANOVA followed by Tukey's multiple comparison tests: * = $p \leq .05$, ** = $p \leq .005$, *** = $p \leq .0005$, **** = $p \leq .0001$. n = 5 WT, 5 Het and 6 cKO brains, from 4 different litters.

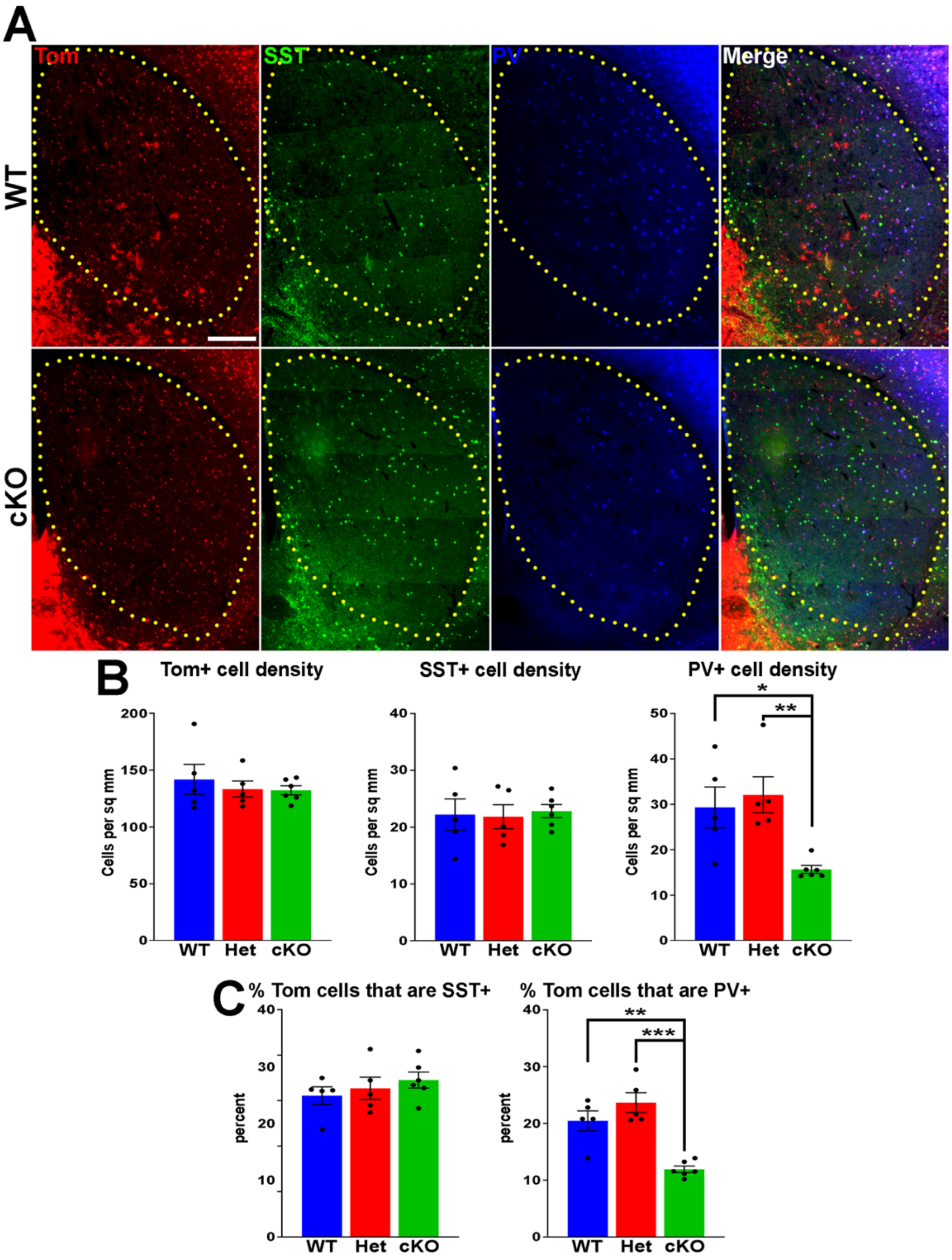

Supplementary Figure 3. Changes in striatal interneuron fate in *Ezh2* cKO mice. A. Representative images through the striatum of P30 *Nkx2-1-Cre*;*Ezh2*;*Ai9* WT and cKO mice stained for SST (green) and PV (blue). Scale bar = 500 μm. **B-C.** Graphs displaying the density of Tom+, SST+ and PV+ cells (**B**) and the percent of Tom+ cells expressing SST or PV (**C**). All stats are one-way ANOVA followed by Tukey's multiple comparison tests: * = $p \leq .05$, ** = $p \leq .005$, *** = $p \leq .0005$. n = 5 WT, 5 Het and 6 cKO brains, from 4 different litters.

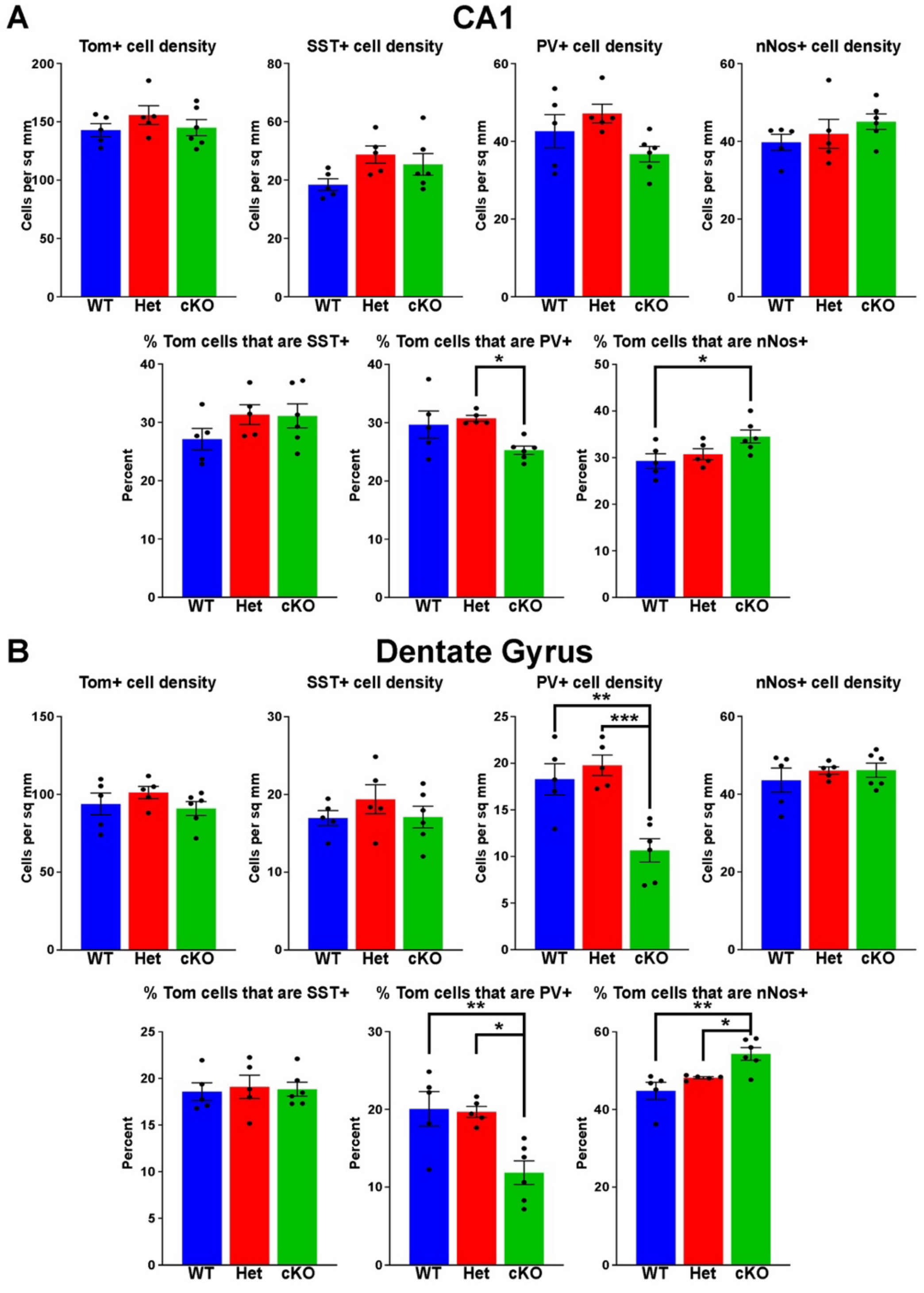

Supplementary Figure 4. Cell fate changes in the CA1 and dentate gyrus of *Ezh2* cKO mice. **A.** Graphs displaying the density of Tom+, SST+, PV+ and nNos+ cells (top) and the percent of Tom+ cells expressing SST, PV or nNos (bottom) in the CA1 region of the hippocampus. **B.** Graphs displaying the density of Tom+, SST+, PV+ and nNos+ cells (top) and the percent of Tom+ cells expressing SST, PV or nNos (bottom) in the dentate gyrus region of the hippocampus. All stats are one-way ANOVA followed by Tukey's multiple comparison tests: * = p $\leq$ .05, ** = p $\leq$ .005, *** = p $\leq$ .0005. n = 5 WT, 5 Het and 6 cKO brains, from 4 different litters.

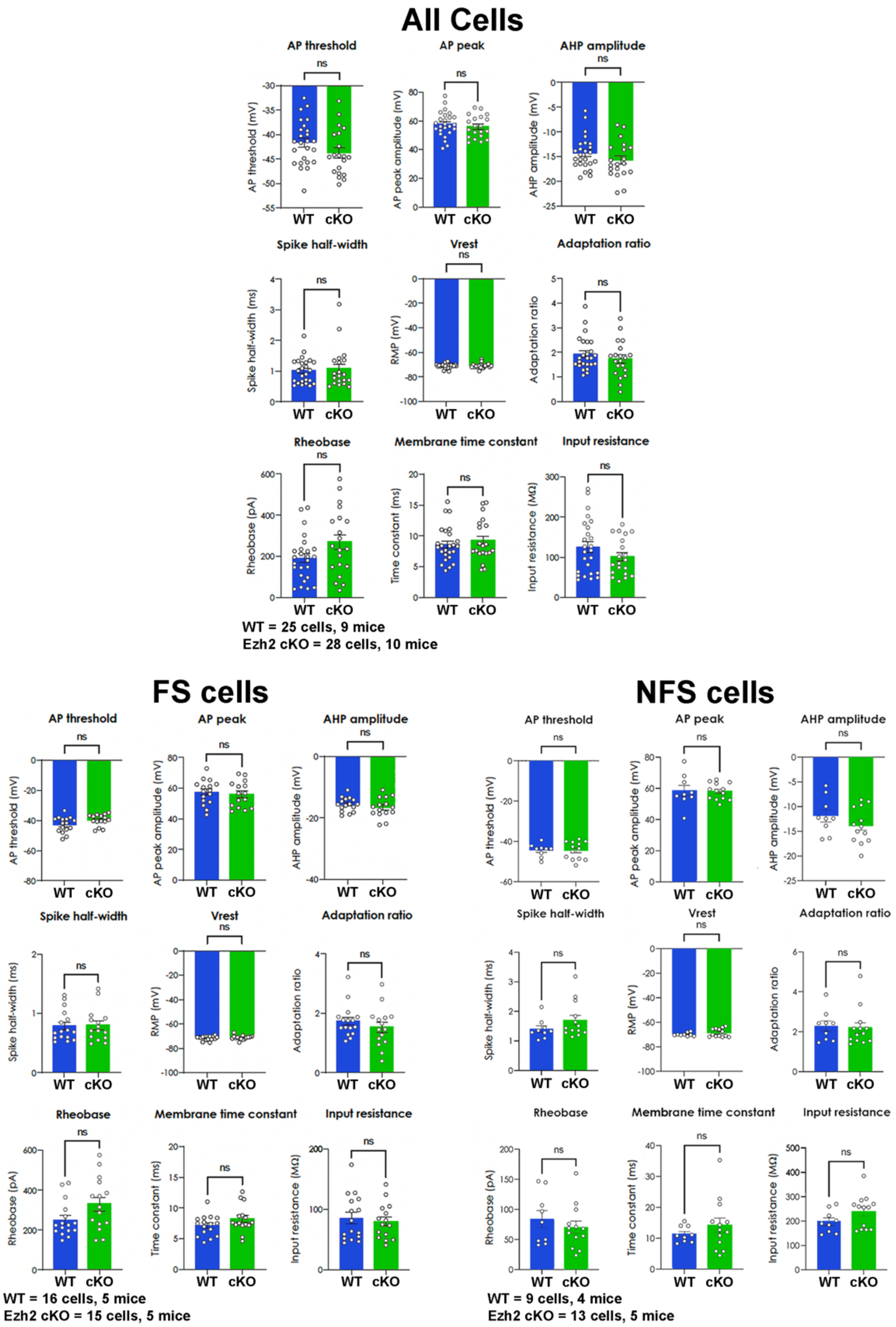

Supplementary Figure 5. Intrinsic physiology properties of MGE-derived cortical interneurons in *Ezh2* cKO mice. Graphs depict intrinsic properties of layer V/VI Tom+ cortical interneurons from P30-40 WT and cKO cells from fast-spiking (FS, lower left), non-fast-spiking (NFS, lower right) and combined FS and NFS cells (top). All statistics are Mann-Whitney test. WT = 16 FS cells from 5 mice and 9 NFS cells from 4 mice; cKO = 15 FS cells from 5 mice and 13 NFS cells from 5 mice.

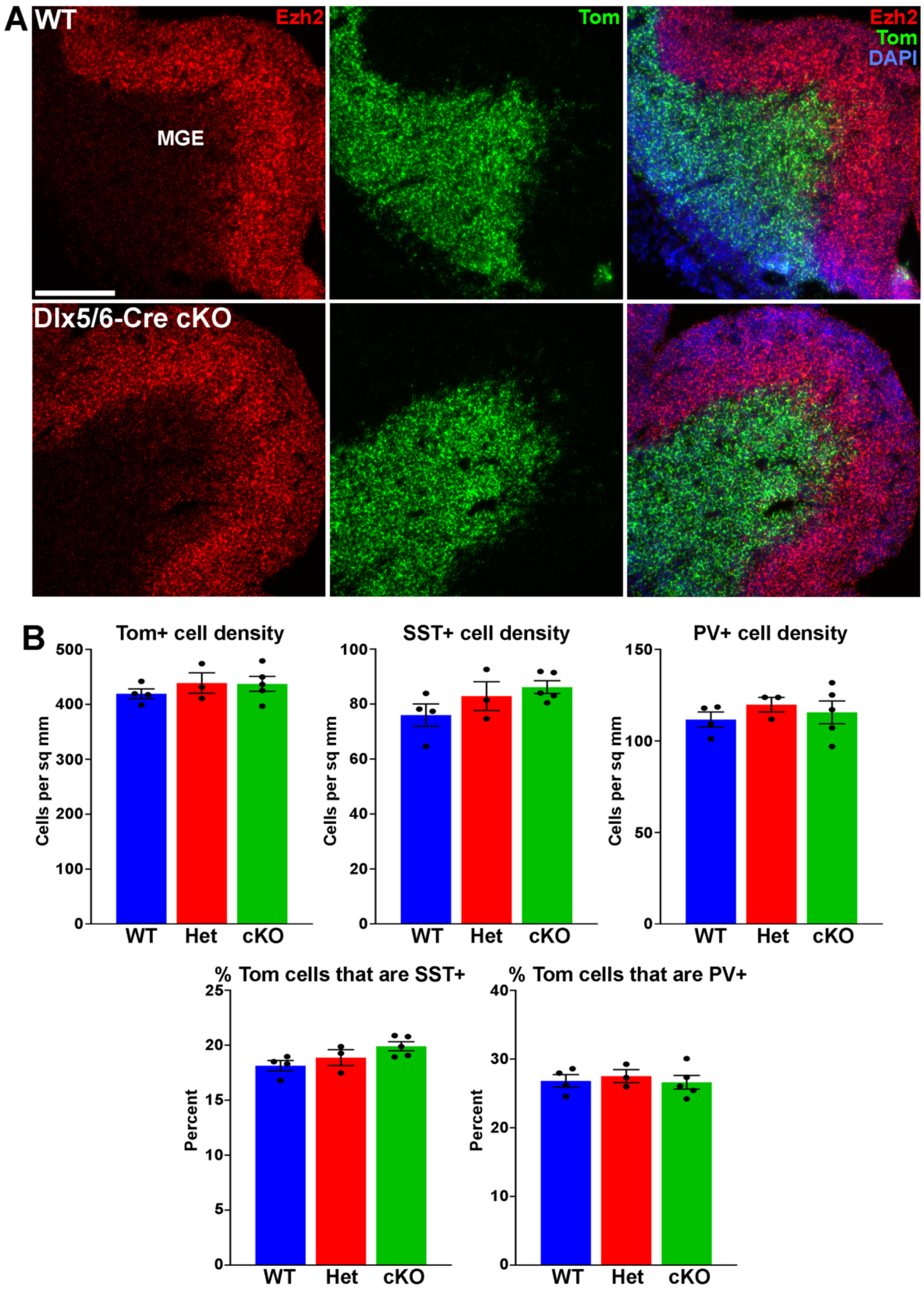

Supplementary Figure 6. Normal interneuron densities and subtype distributions in the cortices of *Dlx5/6-Cre*;*Ezh2* cKO mice. A. *In situ* hybridizations of *Ezh2* (red) and *tdTomato* (green) in the E12.5 MGE of *Dlx5/6-Cre*;*Ezh2*;*Ai9* WT and cKO mice. Scale bar = 200 μm. **B.** Graphs displaying the density of Tom+, SST+ and PV+ cells (top) and the percent of Tom+ cells expressing SST or PV (bottom) in the cortex of WT, Het and *Dlx5/6-Cre* cKO mice. All stats are one-way ANOVA followed by Tukey's multiple comparison tests. n = 4 WT, 3 Het and 5 cKO brains, from 3 different litters.

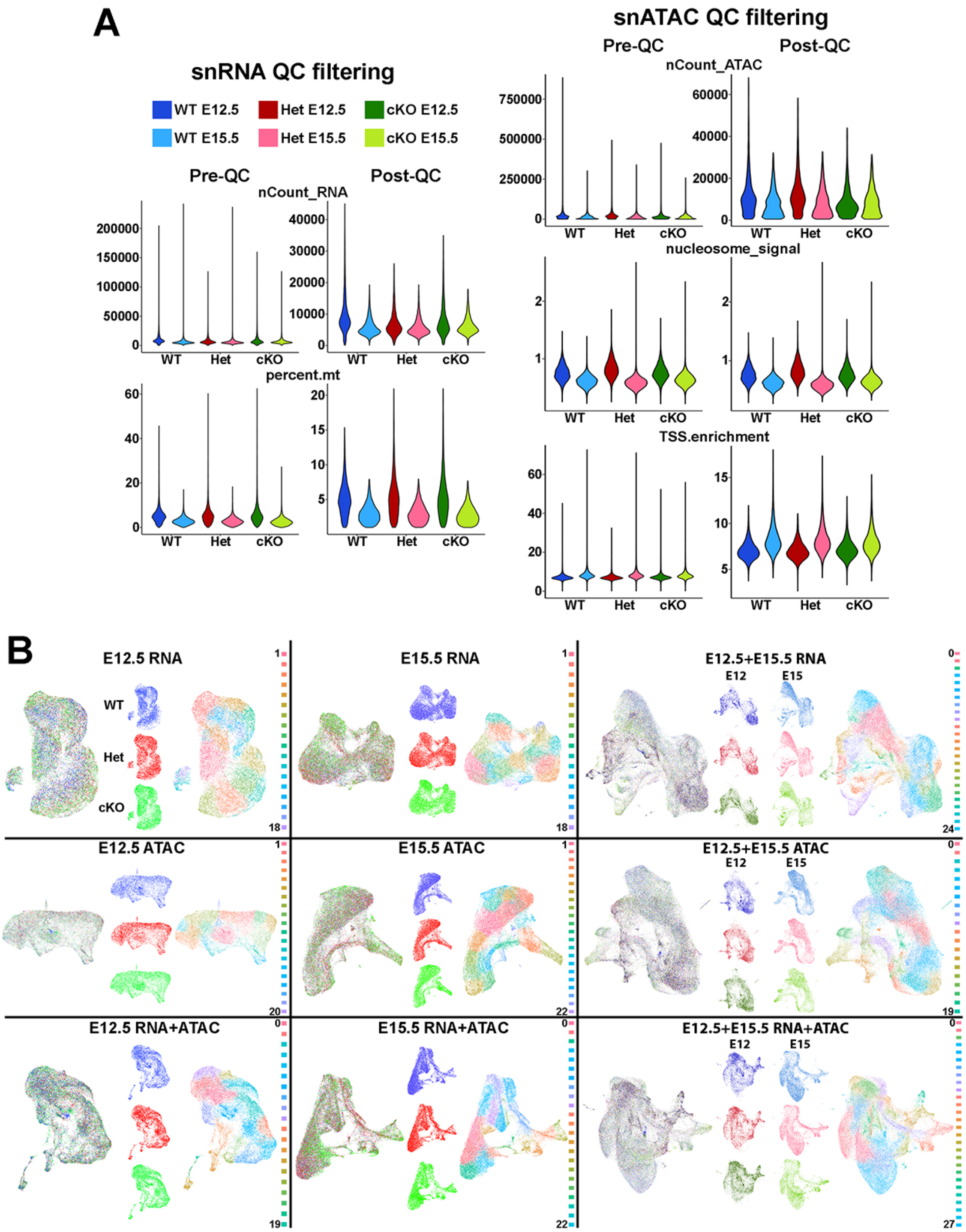

Supplementary Figure 7. Single nuclei Multiome data from *Ezh2* WT and cKO mice. A. Visualization of quality control (QC) metrics before (Pre-QC) and after (Post-QC) filtering of outliers. The number of RNA reads (nCount_RNA), mitochondrial percentage (percent.mt), the number of ATAC fragments (nCount_ATAC), nucleosome signal (nucleosome_signal), and the TSS enrichment score (TSS.enrichment) are shown. **B.** UMAP plots of Multiome data separated by age (E12.5, E15.5 and combined) and modality (RNA, ATAC and integrated RNA+ATAC), with putative cell clusters. WT = blue, Het = red, cKO = green.

Supplementary Table Legends

Supplementary Table 1. E12.5 Multiome data gene list showing average Log_2 fold change (avg_log2FC) and false discovery rate (p_val_adj). Genes with a Log_2 fold change (FC) > ± 0.2 are shaded blue, genes with a false discovery rate (FDR) of $-Log_{10}$AdjustedP < 10^{-6} are shaded yellow, and genes in both groups are in red text.

Supplementary Table 2. E15.5 Multiome data gene list showing average Log_2 fold change (avg_log2FC) and false discovery rate (p_val_adj). Genes with a Log_2 fold change (FC) > ± 0.2 are shaded blue, genes with a false discovery rate (FDR) of $-Log_{10}$AdjustedP < 10^{-6} are shaded yellow, and genes in both groups are in red text.

Supplementary Table 3. E12.5 CUT&Tag data detailing all called peaks. Peaks with a false discovery rate (FDR) < 0.1 are shaded green.

Supplementary Table 4. E15.5 CUT&Tag data detailing all called peaks. Peaks with a false discovery rate (FDR) < 0.1 are shaded green.

Supplementary Table 5. Sequencing depth for all 12 CUT&Tag samples used in this study.

Supplementary Table 6. Sequencing information about RNA and ATAC runs for all 6 Multiome samples used in this study.

Data Sheet 1. Excel spreadsheet containing all cell counts and statistics used in this study.

Data Repository Information

All sequencing data (raw and processed files) generated in this study has been deposited in the Gene Expression Omnibus (GEO) database with the following accession numbers: GSE233190 (CUT&Tag dataset), GSE233153 (single nuclei Multiome dataset, which includes the snRNA/GEX GSE233151 and the snATAC GSE233152 data). No custom code was used in the manuscript, and all computational pipelines are described in the methods. Please contact the corresponding author for more information if needed.
